# Vector-Borne Transmission Imposes a Severe Bottleneck on an RNA Virus Population

**DOI:** 10.1371/journal.ppat.1002897

**Published:** 2012-09-13

**Authors:** Naomi L. Forrester, Mathilde Guerbois, Robert L. Seymour, Heidi Spratt, Scott C. Weaver

**Affiliations:** 1 Institute for Human Infections and Immunity, Center for Biodefense and Emerging Infectious Diseases and Department of Pathology, University of Texas Medical Branch, Galveston, Texas, United States of America; 2 Sealy Center for Preventative Medicine and Preventative Medicine and Community Health, University of Texas Medical Branch, Galveston, Texas, United States of America; Pasteur Institute, France

## Abstract

RNA viruses typically occur in genetically diverse populations due to their error-prone genome replication. Genetic diversity is thought to be important in allowing RNA viruses to explore sequence space, facilitating adaptation to changing environments and hosts. Some arboviruses that infect both a mosquito vector and a mammalian host are known to experience population bottlenecks in their vectors, which may constrain their genetic diversity and could potentially lead to extinction events via Muller's ratchet. To examine this potential challenge of bottlenecks for arbovirus perpetuation, we studied Venezuelan equine encephalitis virus (VEEV) enzootic subtype IE and its natural vector *Culex* (*Melanoconion*) *taeniopus*, as an example of a virus-vector interaction with a long evolutionary history. Using a mixture of marked VEEV clones to infect *C. taeniopus* and real-time RT-PCR to track these clones during mosquito infection and dissemination, we observed severe bottleneck events that resulted in a significant drop in the number of clones present. At higher initial doses, the midgut was readily infected and there was a severe bottleneck at the midgut escape. Following a lower initial dose, the major bottleneck occurred at initial midgut infection. A second, less severe bottleneck was identified at the salivary gland infection stage following intrathoracic inoculation. Our results suggest that VEEV consistently encounters bottlenecks during infection, dissemination and transmission by its natural enzootic vector. The potential impacts of these bottlenecks on viral fitness and transmission, and the viral mechanisms that prevent genetic drift leading to extinction, deserve further study.

## Author Summary

The ability of arboviruses to perpetuate in nature given that they must infect two disparate hosts (the mosquito vector and the vertebrate host) remains a mystery. We studied how viral genetic diversity is impacted by the dual host transmission cycle. Our studies of an enzootic cycle using Venezuelan equine encephalitis virus (VEEV) and its natural mosquito, *Culex taeniopus*, revealed the stages of infection that result in a viral population bottleneck. Using a set of marked VEEV clones and repeated sampling at various time points following *C. taeniopus* infection, we determined the number of clones in various mosquito tissues culminating in transmission. Bottlenecks were identified but the stage of occurrence was dependent on the dose that initiated infection. Understanding the points at which mosquito-borne viruses are constrained will shed light on the ways in which virus diversity varies, leading to selection of mutants that may result in host range changes or alterations in virulence.

## Introduction

RNA virus replication is characterized by a high frequency of mutation, which leads to genetically diverse populations. This diversity is thought to enable RNA viruses to effectively survive within the host (i.e. escape or evade immune responses), to be transmitted, and to potentially adapt to new hosts or vectors. While generating diversity may enhance viral survival, a slight rise above the natural mutation rate can be detrimental, and too little variation has been shown to decrease RNA viral spread and pathogenesis [1], [2]. Thus, RNA viruses must optimize their mutation rate so that enough mutations are generated to enable sufficient diversity for survival and adaptation, yet without producing too many deleterious mutations that can lead to error catastrophe and extinction.

The within-population diversity of RNA viruses is a by-product of their viral RNA-dependent RNA-polymerases (RdRp), as most viruses lack a proofreading domain in this enzyme. This low fidelity leads to a high error frequency for replication of all RNA viruses, which varies between 10^−3^ and 10^−5^ mis-incorporations per nucleotide copied. Genetic diversity acts as a critical determinant of viral evolution by facilitating positive selection (when a mutation confers a fitness advantage and thus produces more progeny), or by genetic drift (fixation of random mutations when populations are small). An extreme example of the latter is termed a bottleneck, which refers to a severe reduction in the population during infection, spread or transmission. Bottlenecks can lead to Muller's Ratchet; because reversion rates are low, asexual populations of organisms that periodically undergo population bottlenecks should tend to accumulate deleterious mutations, unless sex or recombination intervene to allow efficient restoration of the wild-type sequence [3], [4]. The deleterious effect of artificial bottlenecks (i.e. plaque-to-plaque passages) has been demonstrated for many viruses, including the alphavirus *Eastern equine encephalitis virus* (EEEV) [5], [6], [7], [8], [9], [10], [11], [12]. In addition, the limited oral susceptibility of many mosquito vectors to arboviruses (**ar**thropod-**bo**rne viruses) may cause bottlenecks at the stage of initial midgut infection during natural transmission cycles.

Bottlenecks have been identified in many viral systems both *in vitro* and *in vivo*. Studies with *Foot-and-mouth disease virus* (FMDV) by Domingo *et al.* [13] have shown that repeated bottleneck events result in reduced viral fitness. Interestingly FMDV cannot compensate for this reduced fitness, emphasizing the long-term deleterious effects of bottlenecks on virus populations. Additional studies looking at more natural systems have identified bottlenecks when viruses spread between different tissues within a host [14], [15], [16], [17]. In particular much of what we understand about bottlenecks in natural systems comes from studies with plant viruses, which have identified bottlenecks during viral infection and cell-to-cell movement in various plant species [14], [17], [18]. Subsequent studies to identify bottlenecks during insect transmission identified a bottleneck during aphid transmission of cucumber mosaic virus [19], and a separate study quantified the amount of potato virus Y transmitted by the insect vector (∼0.5–3 viral particles), thus confirming the presence of a significant bottleneck during infection of and transmission by insect vectors [20].

Bottlenecks during the transmission cycle could have profound effects on arbovirus evolution, especially on adaptive changes. Experimental studies have demonstrated that the 2-host transmission cycle constrains the ability of another alphavirus, Venezuelan equine encephalitis virus (VEEV) to adapt to new laboratory hosts, presumably due to fitness tradeoffs for efficient infection of mosquitoes and vertebrates. Releasing VEEV from the 2-host cycle via serial passages in a single host facilitates adaptive evolution [21]. This finding has also been confirmed in other arboviruses [22], [23], [24], [25], [26]. Furthermore, bottlenecks during the natural transmission cycle could also limit adaptive evolution if they reduce population sizes to levels where selection cannot function efficiently. Previous work has defined three main infectious transitions or stages during mosquito infection: *(1)* midgut infection, when virions initially infect digestive epithelial cells, *(2)* midgut escape, when the virus must enter the hemocoel to infect secondary target organs and tissues, and *(3)* salivary gland infection, a requirement for oral transmission [27], [28]. Considering that midgut infection and escape often severely constrain the transmission process, they may represent bottlenecks and therefore limit genetic variation after oral exposure of mosquito vectors [21]. In addition, transmission of small amounts of virus in saliva may represent an additional bottleneck during arbovirus transmission [29].

Using virus-like replicon particles, Smith *et al*. [30] demonstrated that VEEV infects only a few cells in the midgut epithelium of the epizootic vector, *Aedes* (*Ochlerotatus*) *taeniorhynchus*. This natural bottleneck may reduce the number of virions that initiate infection within the mosquito, thus reducing genetic diversity in the virus population that eventually spreads to the salivary glands. The presence of a bottleneck in the mosquito vector has also been demonstrated for *West Nile virus* (WNV) in *Culex quinquefasciatus* [31], using similar methods. More recently studies with the mosquito vector *C. pipiens* have also demonstrated the presence of bottlenecks during WNV infection [32].

To further assess the presence and possible importance of bottlenecks on virus evolution and transmission, we used VEEV as a model arbovirus. VEEV emerges periodically to cause major epidemics and equine epizootics, a process that is mediated by adaptive mutations in the envelope glycoproteins that allow enhanced infection of epizootic mosquito vectors or equine amplification hosts [33], [34]. Thus, VEEV epitomizes the ability of RNA viruses to emerge and cause disease via adaptive mutations that lead to host range changes. The VEE antigenic complex of alphaviruses comprises 6 subtypes; of these, ID and IE and subtypes II–VI are enzootic strains that circulate continuously between *Culex (Melanoconion)* spp. mosquitoes and rodents, typically *Sigmodon hispidus* (cotton rats), *Proechimys spp.* (spiny rats) and *Oligoryzmys spp.* (rice rats) among others. For VEEV, the mosquito *C.* (*Melanoconion*) *taeniopus* has been implicated as the enzootic vector of subtype IE strains [35]. This mosquito is highly susceptible to oral infection, and even low levels of viremia in a rodent host can lead to mosquito infection (mosquitoes can be infected after ingesting <5 pfu) and subsequent transmission [36]. This high degree of susceptibility is believed to reflect a long-term evolutionary relationship between the vector and virus in its enzootic cycle.

We therefore hypothesized that the long association of this enzootic vector with VEEV subtype IE transmission has resulted in the high degree of vector infection efficiency. In contrast, a midgut infection bottleneck identified in the epizootic vector *A. taeniorhynchus*, which has only a transient role in VEEV transmission during epidemics, occurs after infection with VEEV subtype IC. In theory the long-term evolutionary relationship of VEEV and its enzootic vector might have limited the presence of bottlenecks during the enzootic cycle. To test this hypothesis, we conducted experimental infections using the enzootic vector to indirectly quantify the sizes of potential bottlenecks during vector infection. We used a mixture of genetically marked clones to follow the VEEV population from artificial bloodmeals through transmission to surrogate rodent hosts using a technique previously described [14], [37].

## Results

### Generation of marked clones

To estimate VEEV populations bottlenecks during infection of the enzootic vector, 10 individually marked VEEV clones were created within the backbone of the enzootic subtype IE strain 68U201 infectious clone. Each clone had 6 synonymous mutations in contiguous codons, except for 68U201-007, which had 5. All mutations were introduced into the nsP2 c-terminus that exhibits high sequence diversity and was therefore assumed to be tolerant of synonymous mutations, and were within 150 nt of each other. Each marked virus and the wild-type (wt; 68U201) were rescued and tested for fitness using several methods to ensure that the markers were relatively neutral: 1) standard replication curves performed in Vero cells and CD-1 mice showed statistically indistinguishable kinetics within one log_10_ of the wt strain 68U201 at all time points sampled (Fig. S1); 2) upon subcutaneous infection of mice, all 10 clones generated viremia titers of >5 log_10_ pfu/ml on day one post infection, indicating that all would be transmissible to mosquitoes (Fig. S2); All mice exhibited comparable weight loss to those infected with the wt (marked clone range: 31–42%, median: 37.5%; wt: 37%), and all died between days 5–8 after infection (data not shown); 3) Infection of a mouse with an equal mixture of all clones showed the presence of all 10 on days 2–5 days; 4) mosquitoes inoculated intrathoracically (IT) with each clone became infected as determined by cytopathic effect (CPE) assays 8 days post inoculation (data not shown), 5) mosquitoes injected IT with equal mixtures of all clones showed equal replication of each 8 days post infection (Fig. S4); and. 6) the survival of clones in mosquitoes following bottlenecks reflected a random process and no particular clones appeared more likely than others to disseminate to the hemocoel or salivary glands, as confirmed by statistical analysis (see below). In total, these data strongly indicate little or no difference in fitness among the marked clones, confirming the usefulness of the presumably neutral markers to assess stochastic viral population events following bottlenecks.

The 10 VEEV clones were validated with the probes and primer sets for real-time RT-PCR. All probes were able to detect the correct clone and did not cross-react with any of the other clones. The probes for clones 005 and 007 gave weaker signals, resulting in ca. 100-fold less sensitivity of these assays compared to the others. We therefore removed these 2 clones from the analysis. To ensure that the elimination of these clones from the analyses would not confound interpretation of our data due to the presence of unaccounted virus, we assayed by real-time RT-PCR a subset of mosquitoes and identified both 005 and 007 in some midguts and bodies that also contained most or all of the other clones, consistent with a lack of sampling bias when clones 005 and 007 were not assayed. Furthermore, to ensure that these clones did not infect or disseminate better than expected, 2 samples negative by real-time RT-PCR for these 2 clones were amplified by standard RT-PCR and deep sequenced. The lack of detectable clone 005 and 007 mutation peaks in the sequences indicated that they were not present. Moreover because these 2 clones were not observed in the brains of the mice infected during transmission experiments or in the saliva of mosquitoes for which those clones had not previously been identified by real-time RT-PCR, we were confident that the removal of these 2 clones does not impact the outcomes of mixed infections or our analyses.

### Identification of a bottleneck during VEEV infection of the *C. taeniopus* midgut

**Figure 1:**
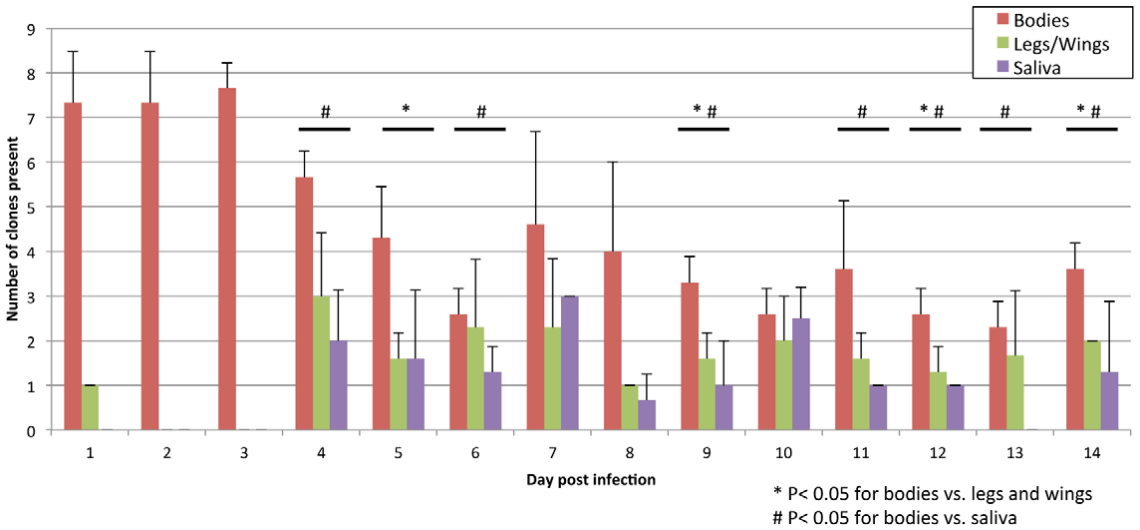
The effect of bottlenecks on a population of marked mixed VEEV clones during experimental infection of *C. taeniopus*. The mean number of clones identified in each tissue, [body, legs/wings (representing the hemocoel), saliva] from mosquitoes sampled daily following oral infection (n = 3). Statistical significance between bodies versus legs/wings and bodies versus saliva was determined by a paired t-test.

Three cohorts of *C. taeniopus* were allowed to engorge immediately following tail vein, intravenous injection of mice with a 200 µl volume containing 6 log_10_ pfu/ml of each marked VEEV clone. Mice were bled before and after mosquito exposure to estimate oral doses. Titers before and after the feed were 6.7 log_10_ pfu/ml (±0) and 6.2 log_10_ pfu/ml (±0.18), or approximately 100-fold higher than would be expected in natural infections, but were designed to give 100% infection of the mosquitoes. Three mosquitoes were sampled daily including bodies as a measure of initial infection, legs/wings as a measure of disseminated infection, and saliva as a measure of transmission potential. RNA was extracted from CPE-positive samples and subjected to real-time RT-PCR to identify the presence of each clone (Table S2). The mean number of clones present in each sample was calculated and the results are presented in Fig. 1. We identified 6–8 clones (mean 7.6) in the bodies of the mosquitoes from days 1–3. There was a decrease in the number of clones present in the bodies at day 4 (mean 3.6) when most blood had been digested or excreted, but clone numbers remained relatively consistent during the remainder of mosquito infections. The legs and wings were consistently positive for viral genomes beginning on day 4 of extrinsic incubation, congruent with earlier time estimates for VEEV dissemination into the hemocoel of *C. taeniopus* [38]. The legs and wings contained between 1–4 clones (mean 1.6) during days 4–14. The presence of VEEV in a single legs/wings sample that was positive on day 1 could be due to a leaky midgut, a phenomenon previously identified during VEEV infections of *C. taeniopus* as well as infections of other mosquitoes by other alphaviruses [38], [39]. Clone content in saliva was also relatively consistently from day 4 (1–3 clones, mean 1.2), although the number of VEEV-positive saliva samples was inconsistent. Because the deposition of saliva into the FBS within capillary tubes could not be visually observed, a lack of salivation could be responsible for some CPE-negatives. While mosquitoes tend to deposit more virus into capillary tubes than into live hosts [29], the salivation technique is intermittent in its success and therefore can underestimate the number of mosquitoes with infectious saliva.

Over 14 days of infection, the number of clones present in mosquito bodies was significantly higher than that present in the legs and wings (p<0.01, by one-way ANOVA with Tukey-Kramer post-test) or in the saliva (p<0.01), indicating that escape from the midgut limited genetic diversity of VEEV populations. However, there was no significant difference between the number of clones in the saliva and the legs/wings (p>0.05) on days 1–14, indicating that the infection of the salivary glands did not detectably constrain VEEV diversity potentially transmitted by *C. taeniopus*.

### Identification of a bottleneck during VEEV escape from *C. taeniopus* midgut

**Figure 2:**
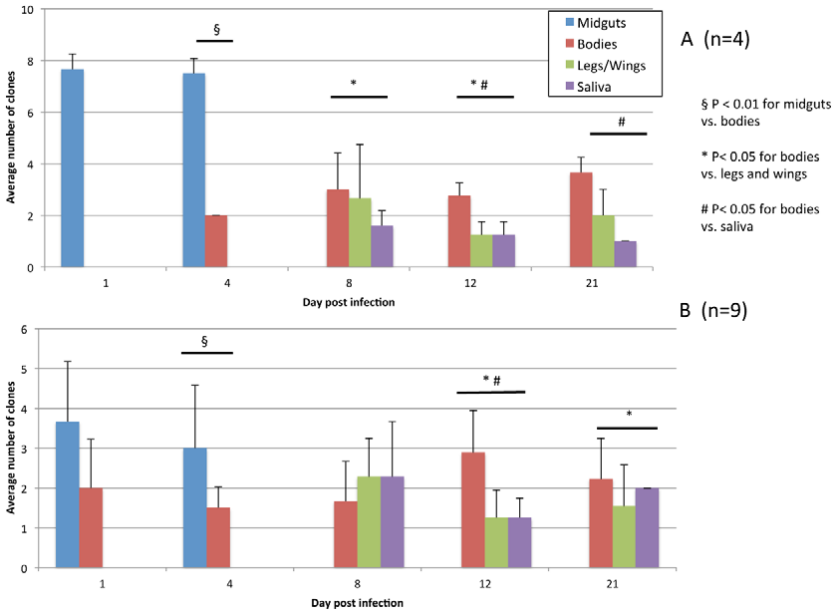
The effect of bottlenecks on a population of marked mixed VEEV clones during infection of *C. taeniopus* with two different starting titers. The mean number of clones present at various time points and in various tissues (midgut, body, legs/wings, saliva) from *C. taeniopus* mosquitoes sampled following oral infection. (A) Mosquitoes infected with a high titer bloodmeal 5.7 log_10_ pfu/ml (n = 4). (B) Mosquitoes exposed to a low titer bloodmeal 4.9 log_10_ pfu/ml (n = 9).

To determine whether a bottleneck occurred during midgut infection, two additional cohorts of *C. taeniopus* were fed with the mixture of clones via an artificially viremic mouse, and midguts were dissected and assayed to assess viral diversity. Artificial viremia titers were 5.7 log_10_ pfu/ml (±0) for the first, high dose cohort and 4.9 log_10_ pfu/ml (±0.47) for the second, low dose cohort. Midguts and bodies were sampled on day 1, midguts, bodies, legs/wings and saliva on day 4, and bodies, legs/wings and saliva on days 8, 12 and 21, with 4 mosquitoes sampled each time point for the high dose cohort and 9 mosquitoes sampled each time point for the low dose cohort. The results of infection and RT-PCR assays are shown in Fig. 2. For the high dose cohort (Fig. 2A), the number of clones present in the midgut was equivalent to that seen in the mosquito carcasses during days 1–4 in the previous experiment (mean 7.6; range 7–8). The midguts were washed to remove residual bloodmeal, so the presence of the large number of clones was likely due to the presence of marked viruses either bound to the midgut epithelium or replicating in the midgut prior to escape into the hemocoel. The bodies, which were positive from day 4 onwards, contained a slightly higher number of clones (mean 2.8; range 2–5) compared to the legs and wings (mean 2.0, range 1–5), which were also positive by day 4. As before, the mean numbers of clones present in the legs and wings and the saliva showed no significant difference.

For the low dose cohort (Fig. 2B) there was a reduced mean number of clones in the midgut compared to the first two experiments (mean 3.7, range 1–6), suggesting that the lower titer of the bloodmeal limited the number of clones that infected initially. Surprisingly, the mosquito bodies from this low dose cohort were positive by day one rather than not before day 4, as in the high dose cohort. This outcome was unexpected because a higher oral dose is expected to lead to a faster midgut replication and escape into the hemocoel. However, there was still a significant reduction in the number of clones present outside the midgut in the mosquitoes sampled on days 4, 12 and 21: midguts vs. bodies (p = 0.004), bodies vs. legs/wings (p = 0.006) or saliva (p<0.001) and bodies vs. legs/wings (p = 0.03), respectively.

### Transmission bottlenecks

**Figure 3:**
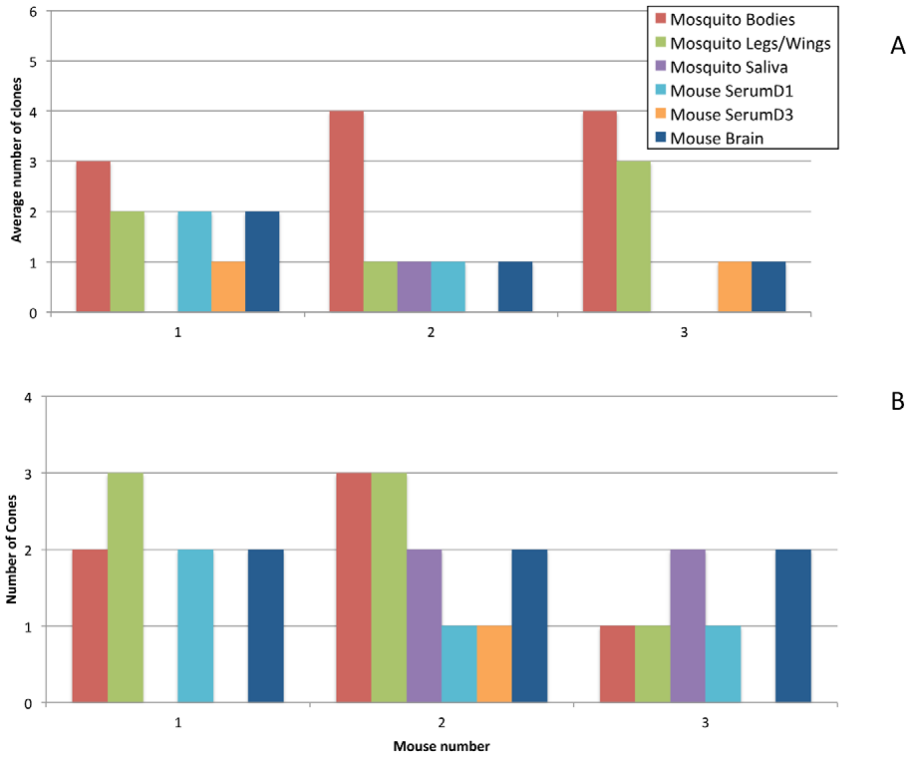
The number of marked VEEV clones observed during transmission from *C. taeniopus* mosquitoes to the vertebrate host. The number of clones present in transmitting mosquitoes and infected mice. (A) Mosquitoes infected at a high dose bloodmeal (5.7 log_10_ pfu/ml) and (B) Mosquitoes infected with a low dose bloodmeal (4.9 log_10_ pfu/ml).

Mosquitoes from both cohorts were allowed to feed on naïve mice at day 21 of the extrinsic incubation period. For cohort one, 10 mice were each exposed to an individual mosquito, and each mosquito was allowed the opportunity to probe and/or engorge for one hour. Mosquitoes were processed immediately after exposure to mice, except that saliva could not be collected from the mosquitoes that engorged. Mice were monitored daily and bled on days one and 3, and the heart, brain, lungs and spleen were sampled on day 6 post infection if the animals showed signs of disease. Of the 10 animals presented to mosquitoes, only 3 showed signs of disease, and their CPE and RT-PCR results are shown in Fig. 3A. Preliminary results from mouse infections with an equal mixture of all 10 clones showed that the clones could be identified in the brain at 5–6 days post infection even if they were not identified in the serum due to sensitivity limitations of the assay (data not shown). Therefore, the number of clones in the brain was used as a surrogate to identify the clones that were transmitted to the mouse. The same 2 clones present in the brain of mouse one were present in the transmitting mosquito legs/wings (representing the hemocoel), indicating no detectible bottleneck during transmission. Similarly, mouse 2 contained only one clone in its brain, which was also the only one present in the legs/wings of the transmitting mosquito. In contrast, mouse 3 exhibited a major bottleneck during transmission; four clones were present in the legs/wings of the transmitting mosquito, yet only one of these was found in the corresponding mouse brain. This bottleneck could have occurred during salivary glands infection, deposition into the saliva, or transmission to the mouse. For the low dose cohort, 15 mice were presented to mosquitoes and again, only 3 developed signs of disease (Fig. 3B). Again there was no difference between the number of clones found in the legs/wings or saliva and the number of clones found in the mouse brain, suggesting no major bottleneck during transmission.

### Identification of a bottleneck during salivary gland infection

**Figure 4:**
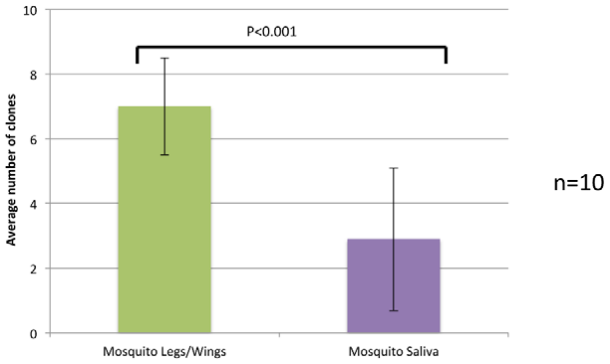
The number of marked clones observed after intrathoracic inoculation of *C. taeniopus* mosquitoes. The mean number of VEEV clones present in the tissues of mosquitoes (legs/wings, saliva) 8 days after intrathoracic infection.

To investigate whether the severity of the bottlenecks at the midgut infection and escape levels masked a subsequent bottleneck during salivary gland infection, mosquitoes were intrathoracically (IT) injected with ∼1–2 µl of a 5 log_10_ pfu/ml VEEV suspension containing all clones to bypass midgut infection and sampled for the presence of virus as described above. Again, because of the limited sensitivity of the assays for clones 005 and 007, these were excluded from the analysis. Figure 4 shows that there was a significantly larger (p<0.001) mean number of clones present in the legs/wings compared to the saliva. These results suggest that, although a salivary gland infection bottleneck was not observable following oral infection, the IT infection that resulted in a greater clone diversity within the hemocoel allowed this bottleneck to be observed.

### Relative amounts of VEEV clones present in midguts and mosquitoes

**Figure 5:**
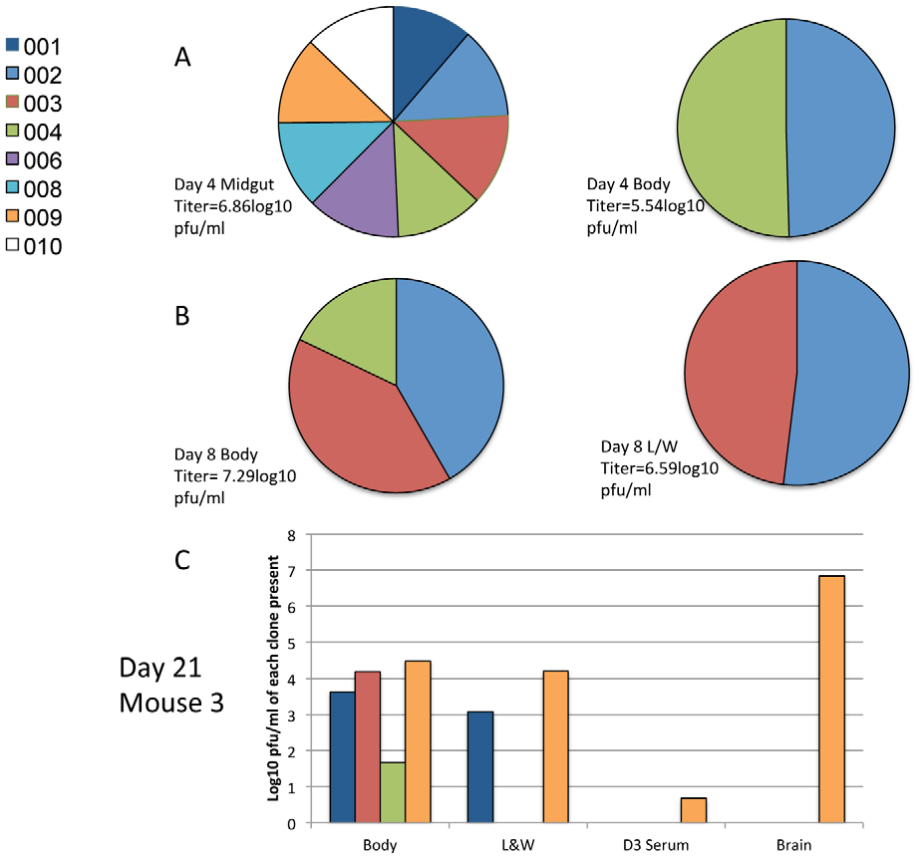
The relative proportions of each individual marked VEEV clone present in a mosquito or during transmission following a high titer bloodmeal. Representative data showing the relative proportions of genetically marked VEEV clones in mosquitoes infected with a high dose bloodmeal (5.7log_10_ pfu/ml) as determined by real-time RT-PCR and extrapolation of infectious titers using standard curves for (A) the midgut and body of a single mosquito on day 4 post infection, (B) the body and legs/wings of a single mosquito on day 8 post infection and (C) the body, legs/wings, day 3 serum and brain for a single mosquito and the mouse infected after a transmission event, respectively. The complete results can be found in Figures S3–5.

**Figure 6:**
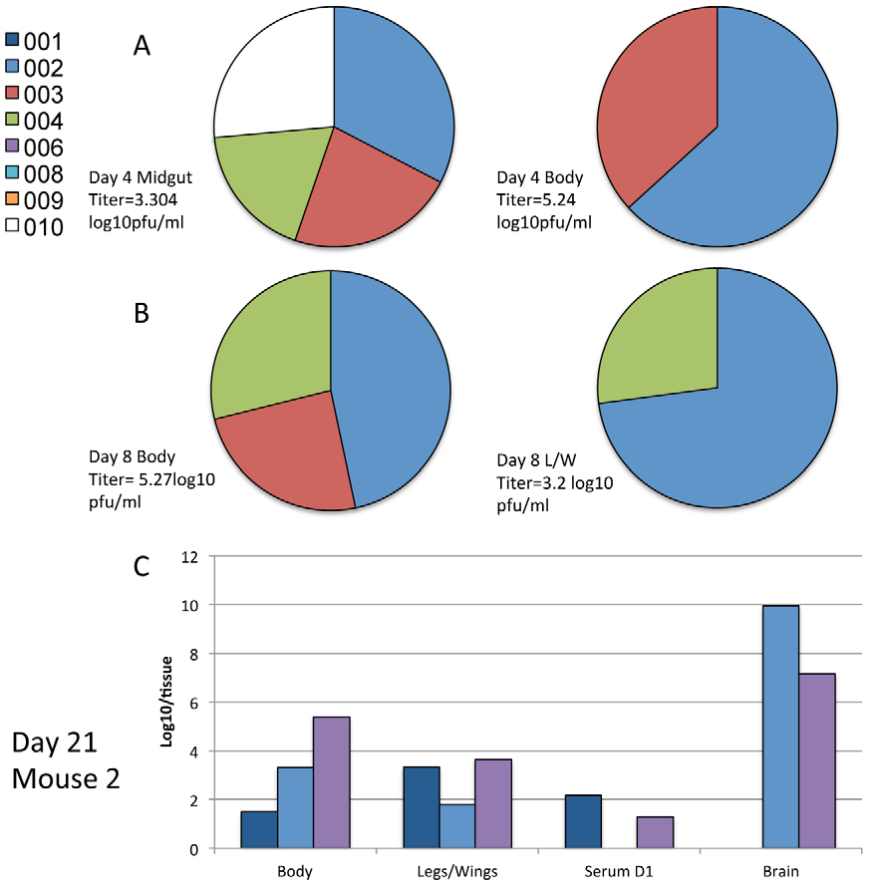
The relative proportions of each individual marked VEEV clone present in a mosquito or during transmission following a low titer bloodmeal. Representative data showing the relative proportions of genetically marked VEEV clones in mosquitoes after infection with a low (4.9 log_10_ pfu/ml) titer bloodmeal, as determined by real-time RT-PCR and extrapolation of infectious titers using standard curves. (A) The midgut and body of a single mosquito, 4 days after infection, (B) in the body and legs/wings of a single mosquito on day 8 post infection, or (C) in the body, legs/wings, D3 serum and brain for a single mosquito and the mouse infected after mosquito transmission, respectively.

To determine the relative quantities of the VEEV clones present at each stage of mosquito infection, we produced standard curves for the real-time RT-PCR assays to estimate infectious titers as previously described [19]. A representative sample of the results is shown in Fig. 5 and 6, and all the results are found in Figs. S3–5. For the high dose cohort, the titers of all 8 clones present in midguts were similar as seen in Fig. 5A. However, the generally smaller numbers of clones in the bodies varied widely in titer, representing major and minor subpopulations. As all clones were present in roughly equal quantities in the midgut, and in the bodies in various ratios, this suggests that there was an equal probability of all 8 clones disseminating from the midgut. No particular clone appeared at high titer consistently in the bodies (Fig. S3), suggesting that little or no selection occurred and that VEEV escape from the midgut was a stochastic process. In all but one case, the clones present in the legs and wings were the same ones present in the highest quantity in the body (see Fig. 5B). Similarly the clones present in the saliva were in general the same ones present at the highest titer in the legs/wings of the mosquito (see Fig. S3). Transmission showed a similar pattern, with the clone/s present in the highest quantities in the legs and wings appearing in the mice. Interestingly, this pattern was seen during progression through all mosquito organs except the midgut (Fig. 5C). After IT inoculation, all 8 clones were present at roughly equal titers within the legs and wings, and the number of clones present within the saliva did not correlate with the relative titers of clones in the legs and wings.

For the low dose mosquito cohort, the clones detected in the midgut were not present in equal quantities (Fig. 6A), suggesting more stochastic variation during midgut infection. However, there was still a reduction in the number of clones disseminating in the body. As observed previously for the transition into the hemocoel (legs/wings), the clones present in the largest quantities invariably disseminated. This was recapitulated in the transition to the legs/wings on day 8 (Fig. 6B), where the 2 clones present in the largest quantities in the body were detected in the leg/wings of the same mosquito. For the low dose transmission experiment, for the first time a clone present in the mouse serum was not seen in the brain. We assume that this animal exhibited a bottleneck such that only 2 out of 3 clones entered the brain. Interestingly, the mosquito transmission of the clones was less consistent than observed in the high dose cohort, suggesting that a greater stochastic element in the initial midgut infection extends to more random dissemination of the clones at various points during the transmission cycle.

### Estimation of the number of founder events

Using F_ST_ statistics, we estimated the number of viral particles initiating infection (N) of the midgut and escaping to initiate infection of the hemocoel. For the high dose cohort, there was no significant bottleneck during infection of the midgut with N estimated to be 1218 (±1318) infectious viral particles. However, for dissemination into the hemocoel, sampled from the legs and wings, the bottleneck N was estimated at 50.9 (±154). In comparison, we observed a strong initial midgut infection bottleneck for the low dose cohort, as the average number of infecting virions was estimated to be 1.9 (±2.6), suggesting that very few infectious virions initiated midgut infection. The average N for dissemination after low dose infection was 1.0 (±1.7), suggesting another strong bottleneck.

### Confirmation of neutrality of marked clones for midgut infection

Neutrality of the clones for infection of the midgut was determined using the ChiSquare test. The number of clones infecting the midguts was compared to the expected number under the assumption that the clonal markers were neutral. For infection of the midgut at a high dose there was no difference between the observed versus expected (χ_2_ = 0.88, DF = 7, P = 0.997). However, for the low dose cohort there was a greater but still insignificant difference between the observed and expected (χ_2_ = 12.1, DF = 7, P = 0.099). This difference for the low dose cohort is likely due to the presence of the bottleneck at the entry into the midgut and therefore the small number of clones analyzed and the resulting high variance. In addition, the presence of all marked clones in roughly equal quantities (mean = 3.42 ±0.74 log_10_pfu/ml according to the qRT-PCR results) again supports neutrality of the markers.

## Discussion

It has been previously postulated that infection of and transmission by mosquito vectors may present bottlenecks for arbovirus populations. To evaluate the potential effects of such bottlenecks during the enzootic transmission cycle of an arbovirus, we used *C. taeniopus* and VEEV subtype IE. The highly efficient infection of this vector by this VEEV strain is believed to reflect a long-term virus-vector evolutionary association. Using a set of marked virus clones, we assessed how often and to what degree bottlenecks affected the number of infectious viral particles transitioning through the mosquito and transmitted to a vertebrate.

Following oral exposure, we identified a major and significant bottleneck when VEEV escapes from the midgut into the hemocoel. Using artificial, intrathoracic infection, we identified a second minor bottleneck at the entry into the salivary glands. This bottleneck was not identified during oral infection, probably because the number of clones in the hemocoel was reduced so severely that a slight drop in the number of clones in the saliva was not apparent. We also examined bottlenecks after oral infection of *C. taeniopus* with 2 different doses differing by about 10-fold, the lower of which probably more closely represents a natural infection. At this lower titer, all 8 clones did not generally infect the midgut. Also, given the lower titer of the bloodmeal it is possible that the blood the mosquito ingested did not contain all the clones as the titer ingested would have been 1.9–2.33 log10 pfu/mosquito. Using F_ST_ statistics we estimated the size of the founder populations for both the high and low dose cohorts. During the high dose there was no major bottleneck upon midgut infection, and all the clones were present in nearly equal quantities. In contrast to the low dose infection, the most severe bottleneck after a high dose oral infection occurred during escape from the midgut into the hemocoel. However, when mosquitoes were infected with a low oral dose, there was a major bottleneck during initial midgut infection, with only approximately 2 infectious virions initiating infection. Following low dose oral infection, there was also a small bottleneck upon dissemination into the hemocoel, with only one clone on average sampled in the legs/wings. Interestingly, given that some clones were present in tissues that had not been positive in assays of the different tissues from the same mosquito, it is likely that some of these clones were present, but at such low quantities that they were not detectable by our methods. Thus, the number of infectious particles initiating the midgut infection following the low dose may be underestimated by our methods.

Experimental infections with rodents collected from VEE-endemic areas in Chiapas, Mexico have indicated peak viremia titers of ca 3–4 log_10_ pfu/ml for VEEV subtype IE. Our results with comparable oral mosquito doses suggest that a major bottleneck during natural mosquito infection occurs at the stage of the initial infection of the midgut. A further bottleneck occurs during dissemination from the midgut into the hemocoel, which leads to infection of the salivary glands.

When mosquitoes transmitted VEEV to mice, no consistent bottleneck could be identified. However, the possibility of a transmission bottleneck cannot be ruled out, especially given the small number of clones that reached the salivary glands after dissemination into the hemocoel and the small number of samples we tested. Further experiments to evaluate potential bottlenecks in the vertebrate host, as suggested by the inconsistency between clone populations in the brain vs. serum of mice, will be required. However, for this study we focused primarily on the number of clones present in the mouse as an indication of the viral population size transmitted from the mosquito. Pfeiffer and Kierkegaard [37] observed a bottleneck when poliovirus crossed the blood-brain barrier. A comparable bottleneck may occur when VEEV enters the brain. However, given that for arboviruses transmission occurs via bloodmeals, bottlenecks at the blood brain barrier are less important than those that affect viremia for continued transmission.

Using a real-time RT-PCR assay and standard curves, we estimated the titers of the individual clones present in mosquito samples for the oral transmission experiments. Clones infecting the midgut were present in similar quantities in the high dose cohort, confirming similar fitness levels for replication in this organ, but not for the low dose cohort. However, after escape from the midgut, the clones were generally present in differing quantities, with no consistency in the relative amounts, indicating that the synonymous genetic markers were indeed neutral; this finding was consistent for both cohorts. With only a few exceptions, the clones that were present at highest titers in the hemocoel transitioned to the salivary glands. This suggests that the clone present at the highest titer in the hemocoel has the best chance of becoming the dominant population in the salivary glands, as expected based on the stochastic nature of genetic drift following bottlenecks.

Genetic diversity is thought to be critical for the survival of RNA viruses, and the presence of a mutant swarm, or intra-host variation, allows them to explore a wider variety of sequence space and therefore adapt to new hosts and new selective pressures [13], [40]. Previous studies with viruses artificially engineered to replicate with a higher-fidelity polymerase demonstrated reduced spread, pathogenesis and fitness in a host when compared with the wt [1], [37], [41]; thus a high level of diversity is imperative for viruses to maintain fitness and robust infections. Given our results suggesting that the mosquito vector is a source of major bottlenecks during the VEEV transmission cycle, the genetic stability of this and other arboviruses and their persistence in their ecological niches is remarkable. Random sampling of viral RNA genomes during population bottlenecks may shift the viral sequence away from the original, fit, or master sequence, thus creating a founder effect. The severe bottleneck at the level of VEEV infection of, or escape from, the midgut could thus have serious consequences for the virus due to the resultant loss in viral genome diversity. Further studies will be needed to determine if and how VEEV is able to restore adequate levels of diversity following these bottlenecks.

Previous experiments with an epizootic subtype IC strain of VEEV and its vector, *A. taeniorhynchus* [30], demonstrated that the midguts of these mosquitoes have only a few cells that are susceptible to initial infection, and thus the entry into the midgut represents a severe bottleneck at infection. Our results with an enzootic VEEV strain and vector underscore potential differences between the evolution of enzootic and epizootic VEEV strains. The presence of all 8 clones at equal quantities in midguts of the enzootic vector, *C. taeniopus* at the high dose, suggests that a larger proportion of its midgut cells are susceptible to infection. Recent studies using VEEV replicon particles also indicate that most if not all *C. taeniopus* midgut cells are susceptible to infection [42]. Since these prior studies used replicon particles to determine the number of cells initially infected, it was not possible to determine whether a midgut escape barrier occurred. Interestingly, for the low dose cohort, the large proportion of susceptible midgut cells did not appear to overcome the bottleneck, as only a very limited number of clones still infected the midgut. Thus, the potential bottleneck and the severity and timing of the initial bottleneck appear to be dependent principally on the titer of the bloodmeal ingested by the mosquito. This has important implications for further experiments to determine the effects on arbovirus evolution of bottlenecks in this and other experimental systems.

A potential conundrum arising from our results and others is that repeated bottlenecks should result in quasispecies constrictions and fitness declines due to Mullers Ratchet [43], as has been demonstrated for viruses and in particular an arbovirus (EEEV) [12]. However, evidence from other alphavirus studies as well as work performed with flaviviruses such as West Nile virus, indicates that arbovirus diversity is maintained even throughout a mosquito infection [44], and most arboviruses are highly stable both genetically and phenotypically in nature [45] and during laboratory passages [46]. Similar work with VEEV is underway in our laboratory. If arboviral diversity is maintained, 3 hypotheses are suggested: 1) the presence of a bottleneck may be compensated by further viral replication and recovery of diversity within the mosquito; 2) bottlenecks are less severe than our VEEV data suggest and there is sufficient diversity retained for maintenance of population fitness, or; 3) many mosquito-rodent VEEV lineages do decline in fitness due to bottlenecks and become extinct, but the large number of such lineages in enzootic or epidemic habitats ensures that some fit lineages remain.

Levels of genetic diversity within natural alphavirus populations have received very little attention, but eastern equine encephalitis virus is known to maintain diversity comparable to other RNA virus populations during natural infections of birds and mosquitoes [47], supporting hypothesis one and possibly 2. Titers of VEEV present in *C. taeniopus* suggest opportunities for the restoration of diversity following a bottleneck event such as midgut escape. The total VEEV titer in the mosquito legs and wings is approximately 6 log_10_ pfu. This suggests that, if only a few virions are responsible for initiation of the hemocoel infection as our data imply, VEEV genetic diversity would be partially restored through the large number of replication cycles, albeit to lower fitness levels if direct reversion is inefficient in restoring fitness as exemplified by Mullers ratchet [43], i.e. restoration of sequence diversity may not necessarily be accompanied by restoration of the high fitness master sequence. The genomic sequencing of different alphavirus isolates collected from the same time and place generally reveal minor differences in consensus sequences, consistent with bottleneck-mediated drift among different transmission lineages and possibly supporting hypothesis 3. Thus the complex interplay of genetic diversity and selection are not really understood, nor has this rate been examined using deep sequencing, which would show the number of mutations generated in the absence of selection. Thus it is unclear to what extent viral diversity could be restored and further experiments using next-generation sequencing will need to be performed to determine the effect of bottlenecks on viral diversity.

In summary, there must be a complicated interplay between the various evolutionary processes to give rise to the viral populations observed in nature. Even within the mosquito, different arbovirus mutations may confer different advantages in critical organs such as the midgut and the salivary glands. In addition, the influence of vertebrate hosts on the maintenance of arbovirus diversity has yet to be determined. Further research to improve understanding of arbovirus evolution will increase insights into the processes that can lead to the emergence of new variants with devastating impacts on human health.

## Materials and Methods

### Ethics statement

This study was carried out in strict accordance with the recommendations in the Guide for the Care and Use of Laboratory Animals of the National Institutes of Health. The protocol was approved by the Institutional Animal Care and Use Committee of the University of Texas Medical Branch.

### Cell cultures and viruses

Vero (African green monkey kidney) and baby hamster kidney (BHK) cells were obtained from the American Type Culture Collection (Bethesda, MD) and maintained in Dulbecco's minimal essential medium (DMEM) (Gibco, Carlsbad, CA) supplemented with 5% fetal bovine serum (FBS), penicillin and streptomycin (100 U/ml). Viruses were rescued from an infectious cDNA clone derived from enzootic, subtype IE VEEV strain 68U201 as described previously and without further passage [30]. The parental 68U201 virus (genomic sequence in GenBank accession no. U34999) was isolated from a sentinel hamster in a sylvatic Guatemalan focus of VEEV in 1968 and passaged once in infant mice and twice in BHK cells prior to cloning [48].

#### Generation of genetically marked VEEV clones

Ten marked VEEV clones were created, each with 5/6 synonymous mutations within 5/6 adjacent codons. Clones were created by joining-PCR and the sequence of each construct was verified prior to cloning into the 68U201 infectious clone backbone. Descriptions of the clones and their mutations are found in Table S1. All viruses were rescued after electroporation into BHK cells as described previously [49].

#### Testing of virus clones

To ensure that the mutations introduced did not compromise viral fitness, individually marked clones were evaluated by comparing their replication in Vero cells, which were infected with an MOI of 0.001 PFU/cell. Samples were taken at every 8 hours for 48 hrs post infection, and titers were determined by plaque assay on Vero cells [50].

#### Mammalian infections

CD-1 mice (Charles River, Wilmington, MA) were infected subcutaneously with 3 log_10_ pfu of virus. Animals were divided into two cohorts and were bled from the retro-orbital sinus on days 1 and 3 or 2 and 4 post-infection. Blood samples were diluted 1∶10 in DMEM supplemented with 10% FBS and stored at −80°C. The mice were weighed daily until they exhibited signs of paralysis, when they were sacrificed. The diluted plasma was plaque assayed on Vero cells to determine VEEV titers [50]. Mosquito transmissions were performed by anaesthetizing mice using sodium pentobarbital (50 mg/kg) before placing them on the screened lids of 0.5 liter cardboard cartons containing mosquitoes, which were allowed to feed for one hour. Mice were then returned to their cages and monitored and bled as described above. The brain, heart, lung and spleen were removed from animals sacrificed on day 6 and stored at −80°C for subsequent analysis. Mice used for oral mosquito exposure were injected via the tail vein with 7 log_10_ pfu/ml of a mixture containing all 10 clones in equal concentrations to generate an artificial viremia of known content (10^6^ pfu of each clone). The mice were anaesthetized with pentobarbital and then bled approximately 5 minutes post injection to estimate viremia titers before being presented to mosquitoes. Mosquitoes were allowed to feed on the mice for 1 hour. Following a terminal blood draw, mice were sacrificed without gaining consciousness. Mouse manipulations were approved by the UTMB Institutional Animal Care and Use Committee.

#### Mosquito infections

Cohorts of *C. taeniopus* mosquitoes (colony originating from Chiapas, Mexico) were sugar-starved for at least 16 hours, then allowed to feed for one hour on mice injected via the tail vein to generate viremia of predictable content, as described above. After one hour, engorged mosquitoes were incubated at 27°C and provided 10% sucrose *ad libitum*. At selected time points mosquitoes were chilled, their legs and wings removed, and then individuals were allowed to salivate for 45 minutes into a capillary tube containing FBS. The midguts were then dissected and the remaining carcass was held separately. Midguts were cut in half and the residual blood was washed out using PBS. All mosquito tissues were placed into DMEM supplemented with 10% FBS, P/S and Fungizone (Sigma-Aldrich, St Louis, MO). For transmission, infected mosquitoes were separated into individual containers and sugar-starved as above. Individual mice were presented to individual mosquitoes and were monitored for feeding for 1 hour, then mosquitoes were sampled as described above. Mice were monitored for signs of disease as described above. Finally, cohorts of *C. taeniopus* were infected via intrathoracic (IT) inoculation using a glass capillary tube heated and pulled to a fine tip, of ca. ∼1–2 ul containing 5 log_10_ pfu/ml of viral stocks containing all 10 clones. Mosquitoes were then provided 10% sucrose *ad libitum* and processed as described above 8 days post inoculation.

### Processing of tissues

All mosquito and vertebrate tissues were resuspended in DMEM supplemented with 10% FBS Penicillin/Streptomycin (for mosquito tissues fungizone (Sigma-Aldrich)) was also added) and homogenized at 26 hz for 5 minutes, then subjected to centrifugation at 3820× *g* for 10 minutes. Saliva samples were subjected to centrifugation at 663× *g* for 10 minutes prior to processing. All samples were tested for the presence of virus by a cytopathic effect assay (CPE). Positive samples were stored at −80°C for subsequent analysis. For saliva samples, supernatants positive for CPE were used in a real-time RT-PCR assay, as the inconsistency in the amount of virus expectorated from the mosquito [29] would have resulted in some samples being below the limit of detection and thus the passaged supernatant was utilized.

### RNA extraction and RT-PCR methods

Virus suspensions were placed into either TRIZOL (Invitrogen, Carlsbad, CA) in a 1∶4 dilution in order to extract total RNA using the manufacturers protocol, or into Buffer AVL (Qiagen, Valencia, CA) and RNA extracted using the column method as per the manufacturers protocol. Real time RT-PCR was carried out using the ABI 7900HT Fast Real-Time PCR system (ABI, Carlsbad, CA). Each reaction was performed using the TaqMan RNA-to-C_T_ *1-Step* kit (ABI) as per the manufacturers instructions in a 10 ul reaction. A list of the primers and the corresponding probes can be found in Table S2. Each probe had a corresponding primer set that was designed to anneal flanking the polymorphic region of each variant. Each sample was tested for each variant individually and each well was run in duplicate. Positive and negative controls were run on each plate and all 10 clones were included as controls to ensure no cross-detection of the other clones by an individual probe. Additionally, we used serial dilutions with titers from 10^6^−10^1^ pfu/ml of the individual clones to create standard curves (data not shown).

### Statistical analyses

Paired T-Tests were used to determine the change in the number of clones from Bodies vs Legs and wings, Bodies vs Saliva and Legs and wings vs Saliva for individual days. Comparison of the differences over the entire experiment was determined by a one-way ANOVA followed by a Tukey-Kramer post-hoc test. The neutrality of the markers was estimated from the data using the ChiSquare contingency table. The size of the bottlenecks was estimated using the F_ST_ statistic as described in Monsion et al (2008) [51]. Briefly, Fst was estimated using the equation
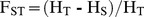
(1)Where H_T_ is the average proportion of clones throughout the entire experiment and H_s_ is the clone proportion within a population. For our experiments each tissue within a mosquito was counted as a population. Using the F_ST_ statistic we were able to estimate the number of clones as a founder population in the mosquito using a second equation,
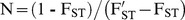
(2)where F'_ST_ is the initial population and F_ST_ the second population, for this experiment the midgut or body and the corresponding legs/wings from the same mosquito respectively. The average N was calculated plus standard deviations.

## Supporting Information

Figure S1 Replication curves performed on Vero cells using all 10 clones individually plus the parental strain 68U201.
(TIF)

Figure S2 Viremia profiles of all 10 clones individually plus the parental strain 68U201 when injected subcutaneously into female 5/6 week old CD-1 mice at 3log_10_ pfu/ml.
(TIF)

Figure S3 The titer of each clone found at each point after oral infection. Mosquitoes were sampled at days 1, 4, 8, 12 and 21. The number of clones in each tissue was identified using real-time RT-PCR. A shows the graphical representation while B shows the data in a dot plot.
(TIF)

Figure S4 The titer of each clone that was involved in the transmission event following oral infection of the mosquitoes from every mosquito tissue and mouse tissue sampled. The number of clones in each tissue was identified using real-time RT-PCR. A shows the graphical representation while B shows the data in a dot plot.
(TIF)

Figure S5 The titer of each clone present after IT inoculation from the mosquito tissues and mouse tissues sampled. The number of clones in each tissue was identified using real-time RT-PCR. A shows the graphical representation while B shows the data in a dot plot.
(TIF)

Table S1 Synonymous mutations introduced into the 10 marked VEEV clones.
(TIFF)

Table S2 List of primers and probes for the real-time RT-PCR assays.
(TIFF)
