# Supplementary figures and images for: Vector-Borne Transmission Imposes a Severe Bottleneck on an RNA Virus Population
Source: PLoS Pathog. 2012 Sep 13;8(9):e1002897. doi: 10.1371/journal.ppat.1002897 (PMC3441635; doi:10.1371/journal.ppat.1002897)

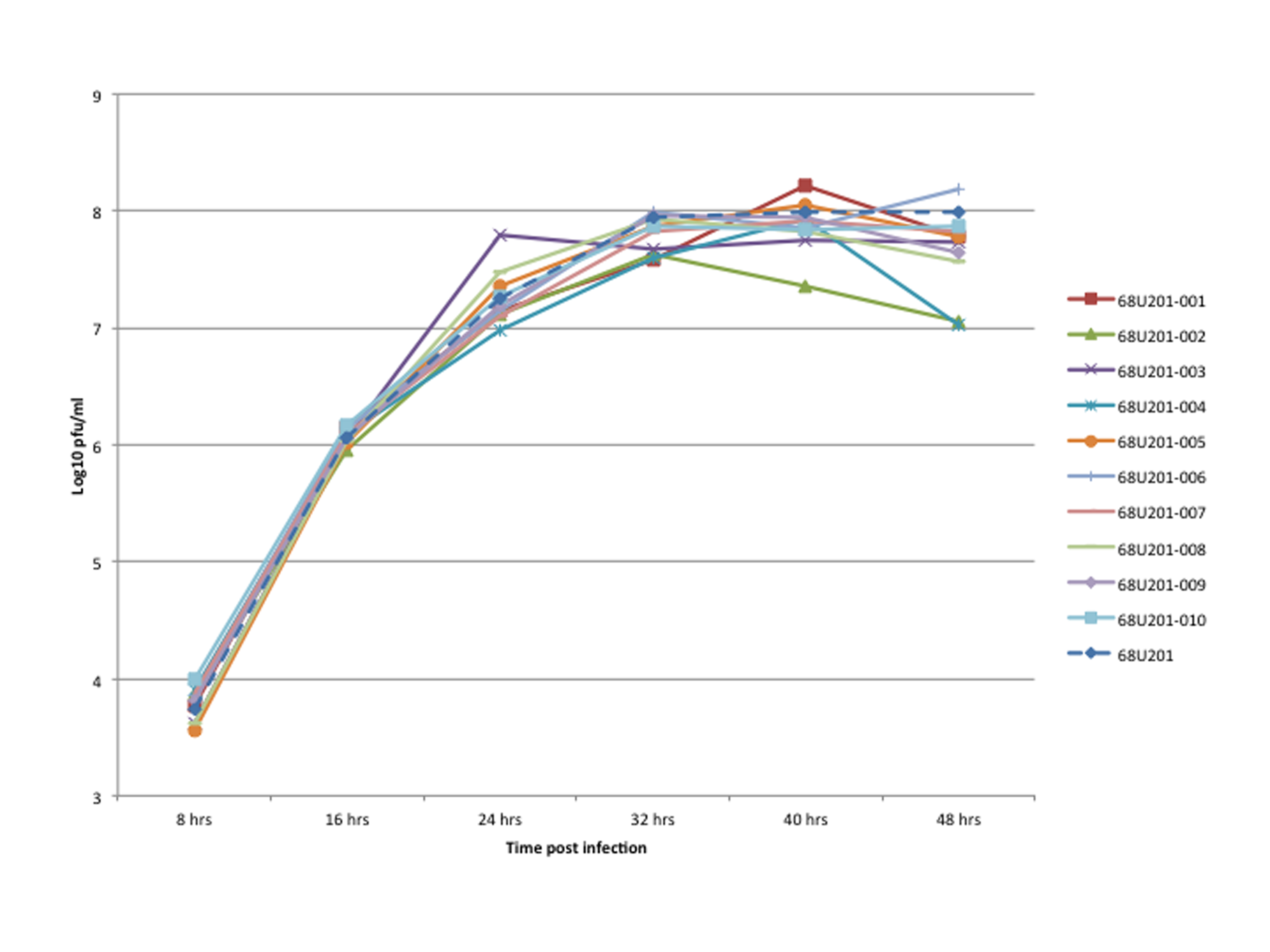

Supplement: Figure S1 — Replication curves performed on Vero cells using all 10 clones individually plus the parental strain 68U201. (TIF) [file ppat.1002897.s001.tif]

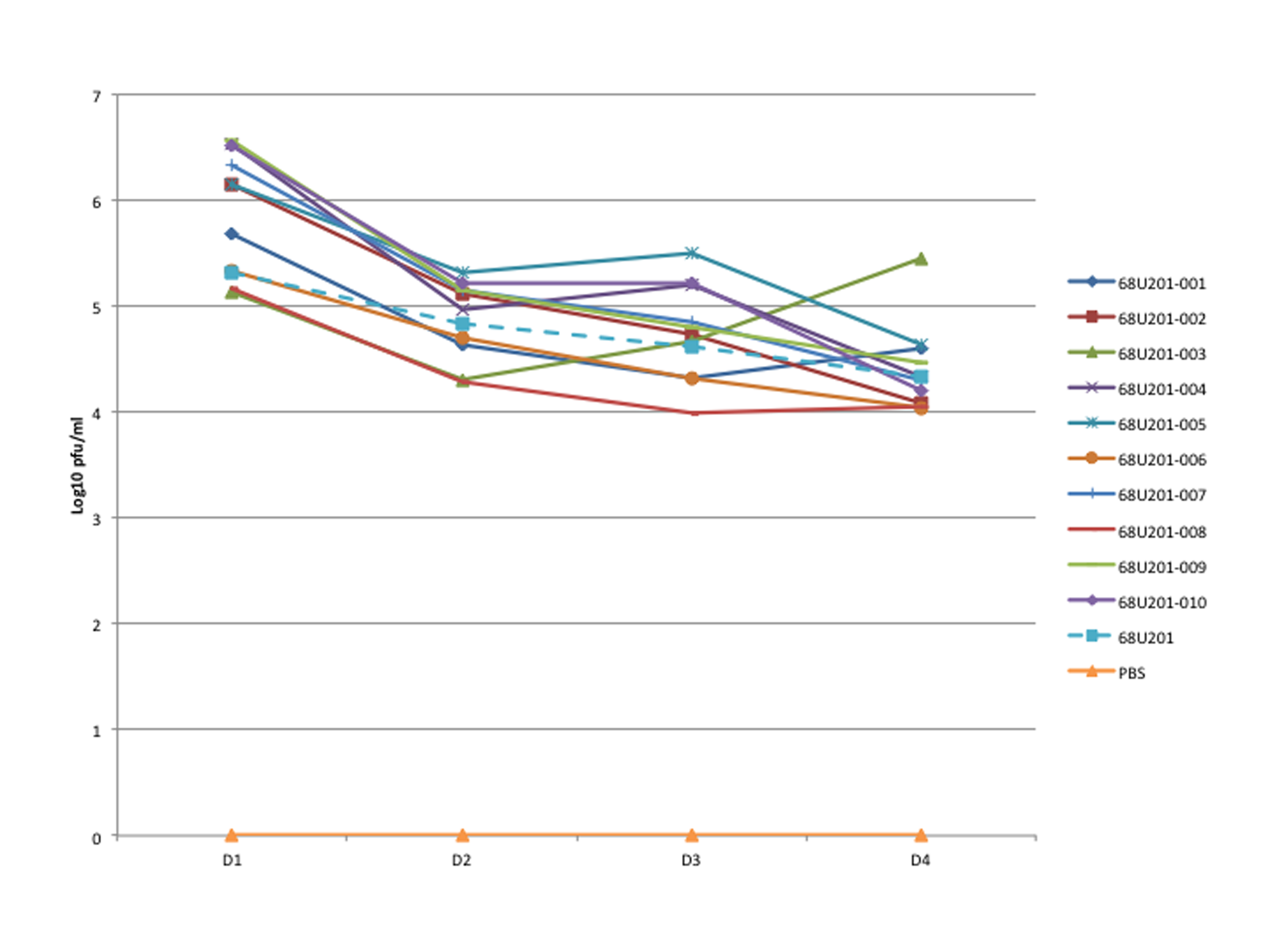

Supplement: Figure S2 — Viremia profiles of all 10 clones individually plus the parental strain 68U201 when injected subcutaneously into female 5/6 week old CD-1 mice at 3log10 pfu/ml. (TIF) [file ppat.1002897.s002.tif]

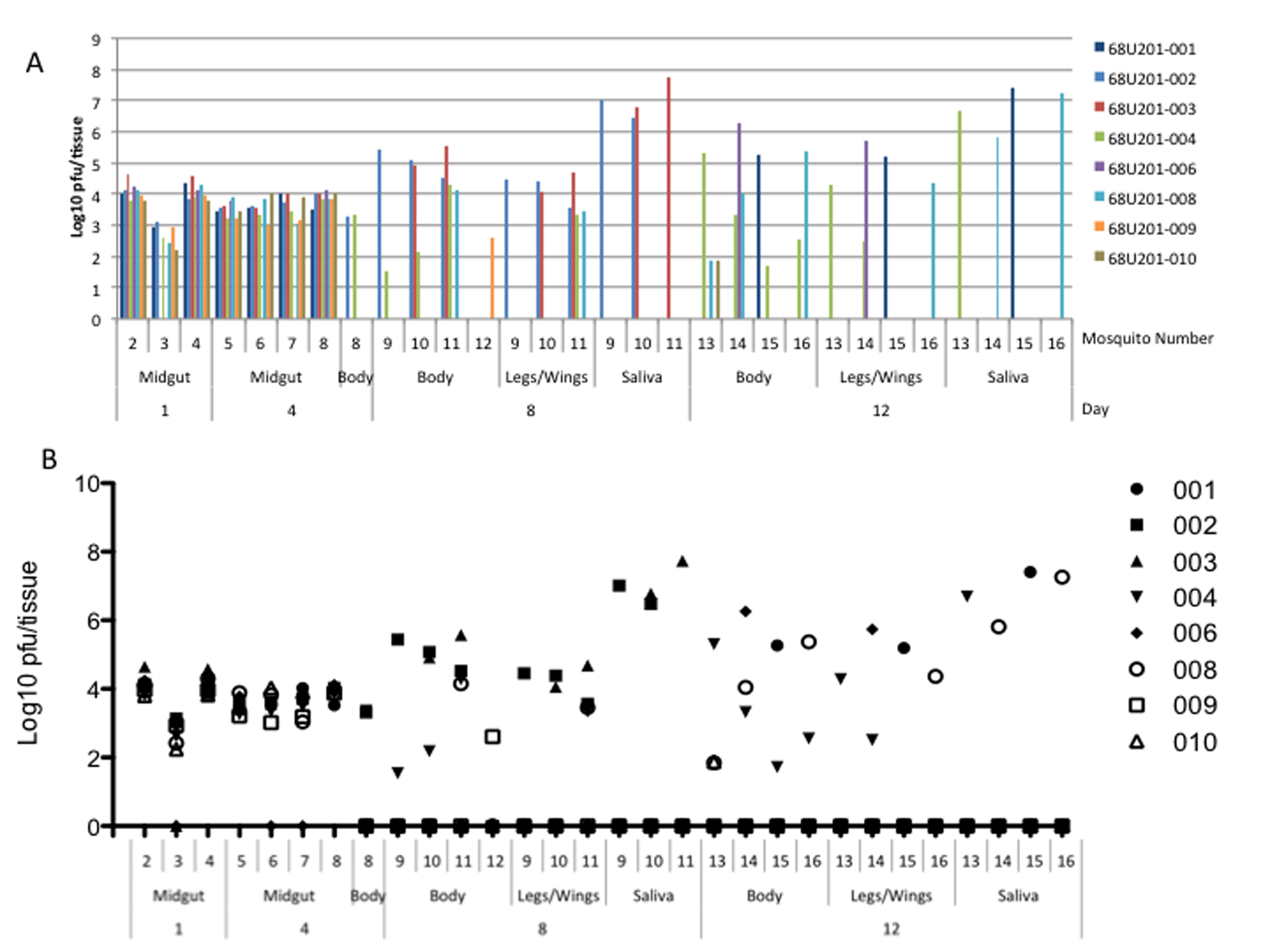

Supplement: Figure S3 — The titer of each clone found at each point after oral infection. Mosquitoes were sampled at days 1, 4, 8, 12 and 21. The number of clones in each tissue was identified using real-time RT-PCR. A shows the graphical representation while B shows the data in a dot plot. (TIF) [file ppat.1002897.s003.tif]

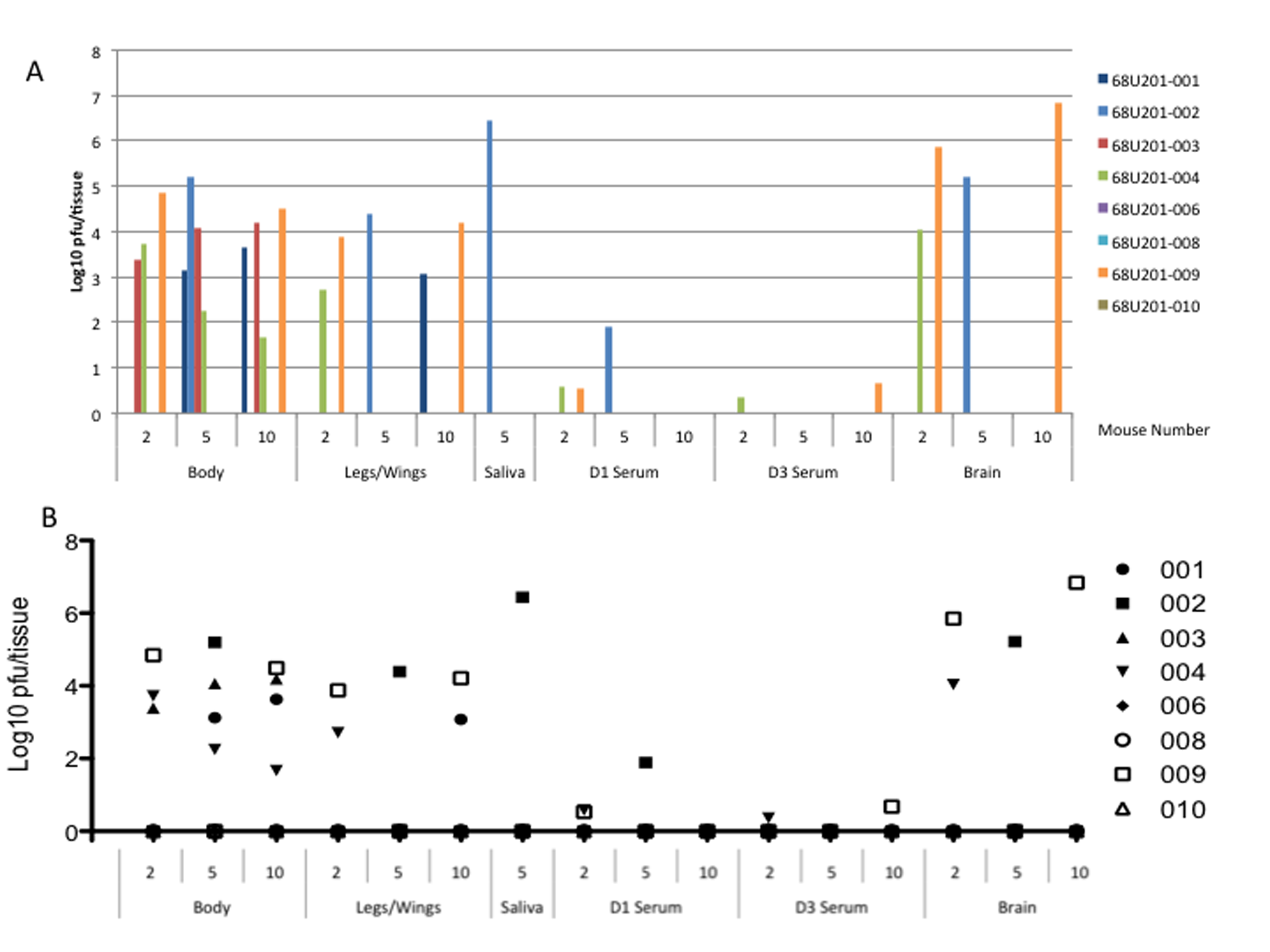

Supplement: Figure S4 — The titer of each clone that was involved in the transmission event following oral infection of the mosquitoes from every mosquito tissue and mouse tissue sampled. The number of clones in each tissue was identified using real-time RT-PCR. A shows the graphical representation while B shows the data in a dot plot. (TIF) [file ppat.1002897.s004.tif]

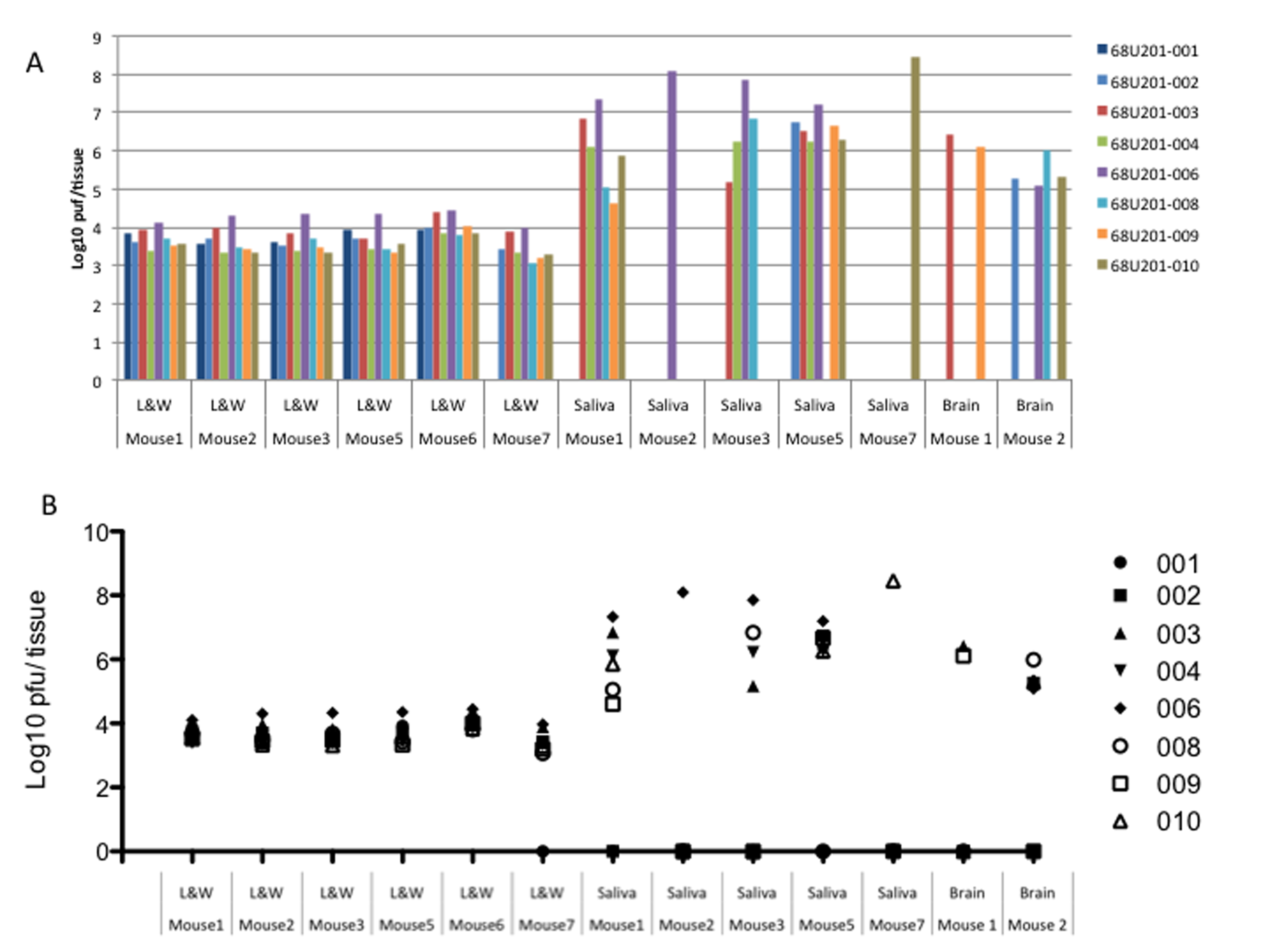

Supplement: Figure S5 — The titer of each clone present after IT inoculation from the mosquito tissues and mouse tissues sampled. The number of clones in each tissue was identified using real-time RT-PCR. A shows the graphical representation while B shows the data in a dot plot. (TIF) [file ppat.1002897.s005.tif]
